# ATG8 protein clustering: a HOTS topic in autophagy

**DOI:** 10.1038/s44319-026-00930-1

**Published:** 2026-09-25

**Authors:** Yongjia Duan, James H Hurley

**Affiliations:** 1 Department of Molecular and Cell Biology, University of California, Berkeley, Berkeley, CA USA; 2 California Institute for Quantitative Biosciences, University of California, Berkeley, Berkeley, CA USA; 3 Aligning Science Across Parkinson’s (ASAP) Collaborative Research Network, Chevy Chase, MD USA; 4 Helen Wills Neuroscience Institute, University of California, Berkeley, Berkeley, CA USA

**Keywords:** Autophagy & Cell Death, Membranes & Trafficking, Microbiology, Virology & Host Pathogen Interaction

## Abstract

A study in this issue of *EMBO reports* shows the ATG8 protein LC3B forms functional nanoclusters on autophagosomal membranes that support autophagy.

**Figure:**
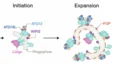

## Teaser

Lipidation of the ubiquitin-like ATG8 proteins is a hallmark of autophagosome biogenesis and has been extensively studied for a quarter century. But there is still more to be learned about what ATG8 proteins really do once they are covalently attached to membranes. Here, combining molecular dynamics simulation, crystallography, super-resolution imaging, and cell biology, Fatihi et al (2026) demonstrate that the ATG8 protein LC3B forms functional two-dimensional nanoclusters on membranes that support autophagy.

Macroautophagy (hereafter, autophagy) is a conserved process for the degradation of various cytosolic materials such as damaged organelles, aggregates, and pathogens. Dysfunction of autophagy is correlated with multiple diseases, including cancer, cardiovascular diseases and Parkinson’s disease and other neurodegenerative diseases (Klionsky et al, 2021). Autophagy can be divided into bulk autophagy and selective autophagy. Both rely on the same core autophagy machinery. In humans, the autophagy core components consist of the ULK1 complex (ULK1C), the class III phosphatidylinositol 3-kinase complex I (PI3KC3-C1), the ATG8 proteins and the ATG8ylation machinery, the phosphatidylinositol 3-phosphate (PI3P)-sensing WIPI proteins, the lipid transporter ATG2, and the lipid scramblase and initiation scaffold ATG9 (Hurley, 2026). Orchestration of these proteins with various lipids ensures the progression of autophagy. Among them, the ATG8 family occupies a central regulatory position. In humans, there are six ATG8 proteins, LC3A-C, GABARAP, and GABARAPL1-2. Upon autophagy induction, all six are conjugated to growing phagophore membrane via reaction with the lipid phosphatidylethanolamine (PE). Membrane-conjugated ATG8s create a platform that recruits a vast array of proteins bearing LC3-interacting regions (LIRs), including the core machinery of autophagy and selective autophagy receptors such as p62 (Lamark and Johansen, 2021). Yet there is still more to be learned about how this ATG8yl-PE platform is organized.

Fatihi et al (2026) began with coarse-grained molecular dynamics (MD) simulations, which provide a “computational microscope” to examine processes that occur on spatial and temporal scales that defy current experimental approaches. They modeled a physiological autophagosomal membrane composition and tested the dynamics of one of the PE-conjugated ATG8s, LC3B. The results demonstrate that LC3B-PE reproducibly forms nanoclusters on the membrane. A basic cluster composed of Arg37, Lys39, and Lys42, forms a pocket that preferentially binds the headgroup of phosphatidylinositol (PI), another prominent lipid involved in autophagosome biogenesis. These properties, with small variations, were also seen for the other five human ATG8 proteins. Thus, at least in silico, ATG8 proteins assemble into high-order nanoclusters enriched in PI lipids.

**Figure 1:**
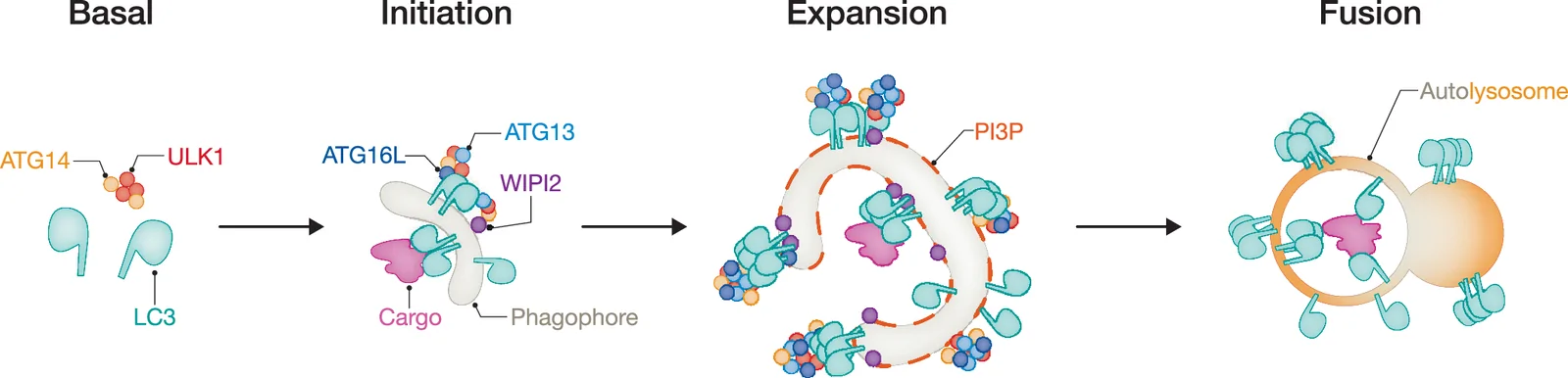
ATG8 clustering and autophagy. LC3B and other ATG8s coalesce in nanoclusters on the growing phagophore, which further drive the organization of the autophagy machinery and its cargo.

Using STORM super-resolution imaging, the authors analyzed clustering in cells. LC3B clusters colocalized with the autophagy core machinery components ATG13, WIPI2, and ATG16L1 (Fig.1). Clusters also colocalized with probes for the lipid phosphatidylinositol 3-phosphate (PI3P), the product of the PI3KC3-C1 complex and another hallmark of autophagosome biogenesis.

Fatihi and colleagues defined two regions that mediate LC3B clustering: alternating polar residues (S87, S90, S91, T93) and hydrophobic residues (M88, V89, V91). Mutations in these regions destabilized the LC3B cluster in MD simulations and altered the clustering pattern in cells. More importantly, they demonstrated that nanocluster formation plays a role in autophagosome motility as well as in the clearance of *Streptococcus pneumoniae* (SPN) bacteria, indicating that LC3B clustering has a physiological function.

This is not the first time the concept of nanoclustering has been introduced in autophagy research. In 2023, Banerjee and colleagues quantitatively analyzed endogenous ULK1 molecules in fed and amino acid starvation conditions. They found that under fed conditions, ULK1 exists in clusters of 30 or fewer molecules, whereas under starvation-induced autophagy, up to 120 molecules of ULK1 were present on the ER. In addition, they found the ULK1 strongly co-clusters with ATG13, ATG14, ATG16, WIPI2, and LC3B under starvation (Banerjee et al, 2023). Recently, we and collaborators found that the ULK1 kinase contains a PVP motif within its intrinsically disordered region (IDR) that can bind to another ULK1 complex subunit, ATG13, in cis or trans, mediating ULK1 clustering (preprint: Duan et al, 2025). This finding provides some evidence in support of a functional role for ULK1 clustering.

Over the past decade or so, the concept of biomolecular condensates has become more widely recognized as a molecular mechanism underlying autophagy (Babic et al, 2026). However, autophagy takes place in the setting of membranes, which are quasi-two-dimensional (2D) surfaces. Concepts of liquid-liquid phase separation in three dimensions are therefore limited in their ability to describe such geometries. In 2025, Zhang et al and MacKinnon articulated the concept of higher order transient structures (HOTS). They demonstrated that in a cardiac-derived cell line, five membrane proteins, including M2 muscarinic acetylcholine receptor (M2R), β1-adrenergic receptor (β1AR), A1 adenosine receptor (A1R), G protein-gated inwardly rectifying K+ channel (GIRK), and Adenylyl cyclase (AC), form small clusters of two or more molecules in a weak-interaction-dependent manner and correlated HOTS formation with GIRK channel activity (Zhang and MacKinnon, 2025). We believe that Fatihi et al’s ATG8 nanoclusters are an example of HOTS at work in autophagy. It will be interesting to see how this concept develops in the autophagy field.
